# Physical activity in an air-polluted environment: behavioral, psychological and neuroimaging protocol for a prospective cohort study (Healthy Aging in Industrial Environment study – Program 4)

**DOI:** 10.1186/s12889-021-10166-4

**Published:** 2021-01-12

**Authors:** S. Elavsky, V. Jandačková, L. Knapová, V. Vašendová, M. Sebera, B. Kaštovská, D. Blaschová, J. Kühnová, R. Cimler, D. Vilímek, T. Bosek, J. Koenig, D. Jandačka

**Affiliations:** 1 Faculty of Education, University of Ostrava, Ostrava, Czech Republic; 2 Faculty of Informatics, Masaryk University, Brno, Czech Republic; 3 Faculty of Medicine, University of Ostrava, Ostrava, Czech Republic; 4 Faculty of Life Sciences, University of Hradec Kralove, Hradec Králové, Czech Republic; 5 VSB-Technical University of Ostrava, Ostrava, Czech Republic; 6 University Hospital of Child and Adolescent Psychiatry and Psychotherapy, University of Bern, Bern, Switzerland; 7 Section for Experimental Child and Adolescent Psychiatry, Department of Child and Adolescent Psychiatry, Centre for Psychosocial Medicine, University of Heidelberg, Heidelberg, Germany

**Keywords:** Air pollution, Environment, Physical activity, Health, Cognition, Neuroimaging, Aging

## Abstract

**Background:**

Air pollution has been linked to increased mortality and morbidity. The Program 4 of the Healthy Aging in Industrial Environment study investigates whether the health and wellbeing benefits of physical activity (PA) can be fully realized in individuals living in highly polluted environments. Herein, we introduce the behavioral, psychological and neuroimaging protocol of the study.

**Methods:**

This is a prospective cohort study of *N* = 1500 individuals aged 18–65 years comparing: (1) individuals living in the highly polluted, industrial region surrounding the city of Ostrava (*n* = 750), and (2) controls from the comparison region with relative low pollution levels in Southern Bohemia (n = 750). Quota sampling is used to obtain samples balanced on age, gender, PA status (60% active runners vs. 40% insufficiently active). Participants are screened and complete baseline assessments through online questionnaires and in-person lab-based assessments of physiological, biomechanical, neuroimaging and cognitive function parameters. Prospective 12-month intensive monitoring of air pollution and behavioral parameters (PA, inactivity, and sleep) follows, with a focus on PA-related injuries and psychological factors through fitness trackers, smartphones, and mobile apps. Subsequently, there will be a 5-year follow-up of the study cohort.

**Discussion:**

The design of the study will allow for (1) the assessment of both short-term variation and long-term change in behavioral parameters, (2) evaluation of the incidence of musculoskeletal injuries and psychological factors impacting behavior and injury recovery, and (3) the impact that air pollution status (and change) has on behavior, psychological resilience, and injury recovery. Furthermore, the integration of MRI techniques and cognitive assessment in combination with data on behavioral, biological and environmental variables will provide an opportunity to examine brain structure and cognitive function in relation to health behavior and air pollution, as well as other factors affecting resilience against and vulnerability to adverse changes in brain structure and cognitive aging. This study will help inform individuals about personal risk factors and decision-makers about the impact of environmental factors on negative health outcomes and potential underlying biological, behavioral and psychological mechanisms. Challenges and opportunities stemming from the timing of the study that coincided with the COVID-19 pandemic are also discussed.

## Background

### Air pollution and health

Life expectancy has been increasing globally with the number of people aged 65 years and above expected to more than double in the next several decades. The projected increase in Europe and North America, specifically, is 48% by the year 2050 [1]. These important gains in life expectancy are outweighed by increasing trends in comorbidity. One important contributing factor to this trend is air pollution. According to the World Health Organization (WHO), 4.2 million deaths worldwide every year are attributed to ambient air pollution, with 91% of the world’s population living in places exceeding WHO air quality guidelines [2, 3].

Air pollution has both acute and chronic effects on human health. Exposure to air pollution has been related with premature mortality and reduced life expectancy [4, 5]. Of the premature deaths estimated to be caused by ambient air pollution, up to 72% occur from ischemic heart disease and stroke, 8% from chronic obstructive pulmonary disease, 14% from acute lower respiratory tract infection, and 6% are related to lung cancers [6]. Interestingly, a recent study examining the relationship between air-pollution and coronavirus fatality concluded that exposure to air pollution may be an important contributor to death related to COVID-19; almost 80% of deaths were observed in 5 regions located in north Italy and central Spain, that also had the highest NO2 concentrations [7, 8]. The negative effects of air pollution have been demonstrated across a range of pollution parameters (e.g., particulate matter - PM_10_, PM_2.5_, nitrogen dioxide - NO_2_, carbon monoxide - CO, ozone, sulphur dioxide - SO_2_, or benzo [a]pyrene) and are thought to occur through a range of physiological mechanisms involving inflammation, oxidative damage, lipid peroxidation, carcinogenic and genotoxic effects [4, 9–11]. Air pollution can however also impact health indirectly through its effect on human behavior.

### Air pollution and behavior

One behavioral factor directly linked to both - exposure to air pollution and health outcomes -is physical activity. Physical activity (PA) brings about many health benefits, however, when performed outdoors in polluted environments, it can increase exposure to air pollutants [11–13]. Additionally, avoidance of PA due to poor air quality may further undermine health or exacerbate disease. An et al. [14] conducted a systematic review and meta-analysis of studies examining the links between air pollution and physical activity. Across seven studies there was a negative association between PA and air pollution (PM_2.5_ or PM_10_) and a positive association was found with inactivity. The results suggested that at least cross-sectionally, air pollution is associated with reduced physical activity.

Data from the Behavior Risk Factors Surveillance Survey (BRFSS) in the US also indicated that the level of inactivity during leisure time correlates positively with ambient PM_2.5_ [15]. In another study of mobile app users in China, people reduced the frequency of outdoor exercise at an increasing degree of air pollution, although there were no differences in average distance and duration of exercise once exercise was initiated [16]. Results of this Chinese study are consistent with a study by Roberts et al. [17], where increased air pollution (PM_2.5_) was linked with increased odds of physical inactivity in the US adult population. Finally, Yu et al. [18] found a positive association between sedentary behavior (number of total weekly hours of sitting) and air quality index (AQI), PM_2.5_, PM_10_, NO_2_ parameters among Tsinghua University in Beijing students. Altogether, these studies suggest that air pollution shows a negative association with PA and a positive association with inactivity or sedentary behavior, although longitudinal investigations are scarce.

Among other behaviors linked to the exposure to air pollutants is sleep. In a representative study of Chinese children aged 2–17 years, it has been shown that exposure to air pollutants increases the odds for sleep disorders and sleep-disorder symptoms (with strongest association for PM_1_ and PM_2.5_) [19]. In a study of college freshmen across a five-year period, increasing concentrations in AQI, PM_2.5_, PM_10_, and NO_2_ were also associated with a reduction in daily hours of sleep [20]. As a behavior, sleep has been linked to many health outcomes, it impacts wellbeing and is also associated with physical activity. Yet there appear to be few studies on the interplay between sleep, physical (in) activity and air pollution [21, 22]. Insufficient moderate PA and sleep duration may also contribute to altered behavioral responses to stress and the development of depressive symptoms [23], underscoring their relevance for mental health and the need to consider links between these behaviors, air pollution and mental health.

### Air pollution and mental health

Air pollutants may play a role in the etiology of psychiatric disorders including depression, anxiety disorders, suicidal behavior and psychoses due to their toxicity on the central nervous system [24–26]. For example, exposure to PM_2.5_ has been associated with an increased risk of depressive symptoms in older women without prior history of depression or cognitive impairment [27] and with both depressive and anxiety symptoms in community-dwelling older adults [28]. Additionally, increases in PM_10_, NO_2_, and O_3_ have been linked to depressive symptoms in the elderly [29]. In a longitudinal, nationally representative study, annual-average measures of PM_2.5_ were significantly associated with increased psychological distress across a 12-year period (after controlling for other sociodemographic and health-related factors) [30]. Yet, a study involving four European cohorts (the Netherlands, Germany, Norway, Finland) and a total of *N* = 70,928 participants failed to find consistent patterns of association between depressed mood and air pollutants across the four cohorts [31], setting off a debate concerning the strength and consistency of the association and the heterogeneous methods used to study it [32, 33]. Intriguing were nonetheless the results of a small study that utilized experience sampling methods and revealed associations between a real-time objective air pollution index (derived from hourly real-time data released by the Beijing Environmental Protection Bureau) and in-time ratings of subjective wellbeing (negative association with affective ratings) and eudaimonic wellbeing (positive association with ratings of meaning and purpose in life) [34]. Both physical (in) activity and sleep covaried with ratings of psychological wellbeing and distress, and both behaviors were linked with risk of mental health disorders such as depression [35–39]. To what extent this “protection” is maintained when PA is performed in air polluted environments requires further study. Especially studies with objective measures of physical (in) activity and/or sleep and those allowing for both long-term and acute (real-time) capture of air polluting parameters on ratings of wellbeing and mental health outcomes are urgently needed [10].

### Air pollution and neurocognitive health

There is a growing body of evidence suggesting that exposure to air pollution might have neurotoxic effects that culminate over time to neuronal damage and loss, and changes in brain structures and function at all ages [40], leading to altered neurodevelopment and cognitive dysfunction, an important intermediate event in the pathogenesis of dementia [41]. Epidemiological studies have shown that air pollution is associated with decreased cognitive performance, neuroimaging correlates and increased risk of cognitive impairment and dementias [42–46]. For example, in a recent study in *n* = 998 older women aged over 73 years long-term PM_2.5_ exposure was associated with a greater decline in immediate recall and learning (but not with other scores of memory), and with progressive atrophy of grey matter, indicative of increased neuroanatomic risk for Alzheimer’s disease [45]. A meta-analysis of four cohort studies from Canada, Taiwan, the UK and the US [47] including more than 12 million elderly aged 50 years and older reported a 3-fold increase in dementia risk with chronic exposure to PM_2.5_ (HR 3.26, 95% CI 1.20–5.31 per 10 μg/m3). Among older US adults, long-term exposure to higher levels of air-pollutants (i.e. PM_2.5_, PM_10_ and NO_2_), were cross-sectionally and longitudinally related to pronounced cognitive decline including memory, executive function and language domains [48]. Furthermore, increasing exposure to PM_2.5_ was associated with lower verbal learning, exposure to NO_2_ with lower logical memory, and O_3_ exposure with lower executive function in a study of cognitively intact middle-aged and older US adults [49]. In the Nurses’ Health Study, higher long-term concentrations of both PM_2.5–10_ and PM_2.5_ were linked to a significantly faster cognitive decline over a two-year period [50]. Studies integrating environmental epidemiology and neuroimaging suggest that white matter, cortical grey matter and the basal ganglia could be targets of air-pollution and that these alterations on the brain-level might underlie the association between air-pollution and cognitive dysfunction in humans, however, the results are inconsistent possibly due to considerable heterogeneity across study populations, MRI methods, pollutants, and methods of cognitive investigation [46, 51]. The exact mechanisms by which air pollutants cause damage to the central nervous system (CNS) are unknown. Several biological mechanisms have been hypothesized with strongest evidence surrounding pathways of systemic inflammation and oxidative stress [52]. Physical activity, on the other hand, has been shown to be protective for brain and cognition and effective in preventing or delaying the onset of cognitive impairment [53]. Regular PA is thought to improve cognitive function [54] and increase neural plasticity through modifications in vascular physiology, reduction of neuroinflammation and cerebral oxidative stress, and through increases of serum brain-derived neurotrophic factor (BDNF) induced by exercise [55, 56]. There is some evidence suggesting that regular exercise in highly polluted air might not result in the same psychological and neurological benefits that are observed in non-polluted environments [57]. However, whether regular exercise in highly polluted air may actually negatively impact cognition as well as brain structure and function, remains unresolved. Importantly, the optimal level of regular PA that is beneficial from a neurological perspective and mental health may differ depending on air quality.

## The present study

Unfortunately, the interpretation of existing findings linking air pollution to mental or neurocognitive health remains limited given the majority of retrospective study design, varying observational periods and methods, and failure to account for confounding factors such as age or health status [58]. Indeed, the strongest associations are often found in individuals with multiple risk factors (e.g., lower socio-economic status (SES), poorer health status or a history of depression) [28, 59]. Other limitations of studies targeting behavioral outcomes such as PA stem from the fact that they mostly rely on self-reports, retrospective assessments of behavior, which can be biased, and collect averaged annual data on air quality, thereby lacking the dynamic sensitivity to detect acute/short-term changes while also evaluating long-term trends. Important technological advances have taken place in the last several decades, allowing for near real-time assessments of both behavior and psychological outcomes. With smartphone use becoming ubiquitous [60] and the availability of commercial wearable technologies ever increasing, there has never been a more opportune time to make use of these tools to assess the interplay between behavior, psychological states and air quality parameters. Combined with robust laboratory-based methods for the comprehensive assessment of health status of individuals, these methods, sometimes referred to as Dynamic Real-time Ecological Ambulatory Methodologies (DREAM) [61], may help to significantly improve the quality of assessment protocols in prospective studies, addressing links between air-pollution, health, and wellbeing.

Herein, we describe the behavioral, psychological, and neuroimaging aspects of the study protocol from Program 4 of the Healthy Aging in Industrial Environment (HAIE) project, including how mobile technology is utilized to collect real-time data on air quality, behavior, and daily psychological functioning. HAIE consists of four research programs that are interlinked using the same selected population groups study the impact of individual and environmental determinants of health, wellbeing and healthy aging. Program 4 of the HAIE project (4HAIE) specifically focuses on the links between air pollution, biomechanical, physiological, psychosocial, and sociodemographic variables (and their interaction) on the incidence of running-related injuries, physical (in) activity, health and quality of life. Herein, we describe the protocol for the assessment of behavioral, psychosocial and neurocognitive outcomes as part of the study, including key domains of interest in the relationship between air pollution, selected behavioral parameters, psychological functioning and neuroimaging correlates.

## Methods

### Study sample and recruitment

**Fig. 1:**
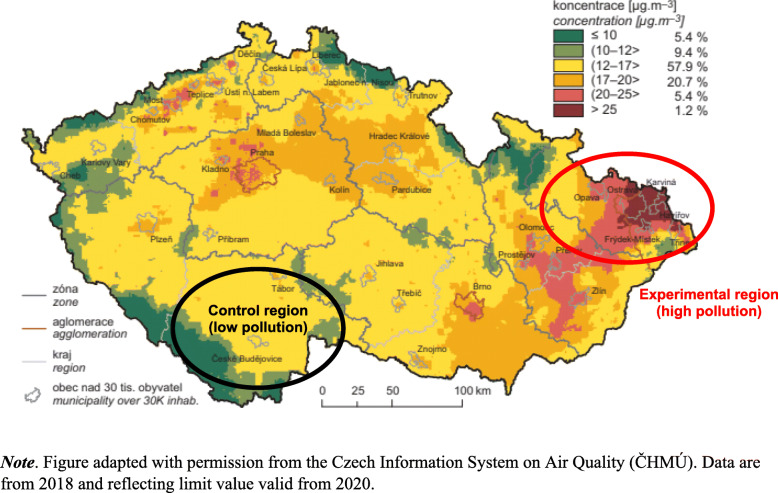
Field of annual average concentration of PM2.5 in 2018 indicating limit value valid from 2020_._ The figure was adapted from publicly available source with author permission (Air Quality Information System. Air Pollution in the Czech Republic in the year 2018. 2018. https://www.chmi.cz/files/portal/docs/uoco/isko/grafroc/18groc/gr18cz/PrilohaII_CHMU2018.pdf. Accessed 22 Jun 2020)

The primary objective of the 4HAIE project is to establish prospective study cohorts to assess the effects of air pollution on health and quality of life across the lifespan. The cohorts are recruited from two different regions within the Czech Republic that differ in air quality. The highly polluted Moravian-Silesian Region (MSR) of the Czech Republic (where life expectancy is about 2 years lower compared to the Czech Republic average [62] and the control South Bohemian Region (SBR) with comparatively low levels of air pollution (See Fig. 1). The Moravian-Silesian region has been long term European hotspot of ambient benzo [a] pyrene and particulate matter pollutants [63]. Exposure to benzo [a] pyrene air pollution in MSR (Ostrava-Radvanice) has been the highest in the European union for a decades [64].

For the 4HAIE study, specifically, the recruitment of study participants was commissioned to a professional social science research and marketing company selected through a publicly advertised tender. The sample is collected using quota sampling based on location, age, gender, and PA status (active-runner vs. inactive) (for details see Table 1).

**Table 1:** Recruitment quotas based on age, region (SBR = South Bohemian Region, MSR = Moravian-Silesian Region) and activity status

Age group	Runners (60%)		Inactive Controls (40%)	
	SBR	MSR	SBR	MSR
18–25	90	90	60	60
> 25–35	90	90	60	60
> 35–45	90	90	60	60
> 45–55	90	90	60	60
> 55–65	90	90	60	60
Total N	450	450	300	300

A total of *N* = 1500 participants aged 18–65 years will be recruited for this study. Of these, *n* = 900 (60%) will be physically active runners and *n* = 600 inactive controls. The runners and inactive controls will be split evenly between two regions: the experimental highly-polluted industrial region (Moravian-Silesian Region – with the exception of the district of Bruntal and selected counties in the district of Frydek Mistek) and the control low-pollution level region (South Bohemian Region). The selected sample size was determined as the largest sample size feasible, allowing for stratification of the cohort by age group and analysis of outcomes targeted by the interdisciplinary teams involved. No formal unifying power analysis was however conducted.

Participants are being recruited from the community using various approaches including in-person recruitment through agency workers, online (using social media, job portals and other websites) and offline advertisements (billboards, newspaper ads, flyers). Recruitment activities focused specifically on runners have been conducted, such as presentations and information booths at running races, through running clubs, and at other relevant community events. Participant recruitment began in February 2019, with the first participants undergoing baseline testing in April 2019. To date (November 2020), *n* = 1012 participants have been recruited and underwent baseline assessments, of which *n* = 360 completed the entire 12-month study. Baseline testing was expected to be completed by the end of 2020, with 12-month intensive monitoring of all participants concluding by the end of 2021. Due to restrictions imposed in the Czech Republic as part of combating COVID-19, testing was interrupted between March 13–April 30, 2020, extending the anticipated recruitment and baseline testing period until June 2021. Up-to-date status of recruitment progress (along with the sociodemographic composition of the sample) can be followed on the study website https://www.4haie.cz/.

### Participant incentives

Participants receive small financial compensation for the baseline laboratory assessments of 40EUR for MSR participants and 70EUR for SBR participants (to compensate for the additional travel time from SBR). Free transportation is provided for SBR participants. Upon completion of the baseline testing, participants also receive results from several baseline assessments (e.g., graded-exercise test, body composition) and a gift bag. Throughout the 12-month monitoring period all participants are eligible for a lottery to win one 185EUR gift card every month. The number of entries into the lottery is based on completing different aspects of the study and reflects the participant’s compliance in the given month. Upon study completion the participants receive detailed personalized feedback on their behavior, survey responses and air pollution data in the form of a final study report.

### Inclusion and exclusion criteria

All participants had to be non-smokers between the ages of 18–65 years at the start of the study. Active runners had to meet public PA guidelines (150 min/week moderate or 75mins/week vigorous PA or an equivalent combination of the two as per WHO recommendations; [65]), be running at least 10 km per week in the last 6 weeks and plan to continue running for the next 12 months. Inactive controls do not meet the public PA recommendations but are capable of normal PA including running (i.e. no physician diagnosed restrictions). Both runners and inactive participants must live in the region for the last 5 years and should have no plans to move outside the region in the next 12 months. All participants are required to own a smartphone (with Android ≥5.0 or iOS) and have internet access (through WiFi or mobile data).

Main exclusion criteria include being a smoker, having an acute health problem (e.g., illness) or injury/surgery/pain preventing normal PA (within the last 6 weeks) and having contraindications to magnetic resonance imaging (MRI) or dual-energy x-ray absorptiometry (DXA) (e.g., pregnancy, radiological examination in the last 7 days using iodine / barium contrast agents, pacemaker, radioactive body, surgical staples, insulin pump, cochlear implant, other metal implants and foreign bodies such as shrapnel, etc.).

### Study timeline and procedures

Interested participants complete screening online and by telephone. Eligible participants are then invited to complete first two baseline questionnaires from home (through the Qualtrics online survey platform) and are scheduled for a 2-day laboratory assessment. Upon arrival to the laboratory (up to 4 participants are tested each day), informed consent is administered in person and participants obtain a hard copy of the signed informed consent to keep for their records. The 2-day laboratory assessments include a series of physiological, biomechanical, anthropometric, cognitive and MRI assessments (also described in other protocols currently under review). Participants also complete two additional online questionnaires while in the laboratory and undergo in-person training in study procedures. During this training, lab staff install mobile apps (a custom-made survey app and the Fitbit fitness app) on the participants’ smartphones. Participants are familiarized with the study protocol, the apps, and receive detailed instructions for using a fitness bracelet (Fitbit Charge 3). The orientation to the study procedures, apps, and fitness bracelet takes approximately 40 min. Cognitive testing and neuroimaging take place in the morning after breakfast on day 2. Cognitive assessment is conducted in a quiet room and takes approximately 45 min; neuroimaging takes about 10 min. The study procedures have been approved by the Ethical Committee of the University of Ostrava.

### Questionnaires

As part of the study, participants complete a series of four separate baseline questionnaires: two at home prior to arrival to the laboratory (Socioeconomic and Psychological Survey) and two sets of questionnaires (Physical Activity Survey and Biomechanical Survey) in the laboratory.

The Socioeconomic Survey, prepared in collaboration with the study partner of Program 1 of the HAIE project (Faculty of Medicine at the University of Ostrava), consists of questions about sociodemographic factors, basic lifestyle factors, risk perception, health status, and quality of life. The completion of the survey takes approximately 40 min. The Psychological Survey includes questions concerning psychological protective factors (such as social support) and risk factors (such as perceived stress, depressive symptoms, or neuroticism). Questions about sleep are also included. The completion of the survey takes approximately 30 min. The Physical Activity Survey completed in the laboratory is composed of questions about physical activity, sedentary behavior, motivational and psychological factors associated physical (in) activity and running history. The completion of the survey takes approximately 45 min. In the Biomechanical Survey (20 min) participants are asked about injury history and pain. A detailed summary of the questionnaires and measures used in this study can be located on the study website https://www.4haie.cz/data/.

At month 6 and month 12, participants are asked to complete a subset of selected measures online from home again. The completion of each of the follow-up surveys takes approximately 45 min.

### Hair samples

Until a few years ago, cortisol has been analyzed only from blood, saliva or urine to assess acute or short-term circulating cortisol concentrations [66]. Whereas these acute and transient cortisol reactivity are part of the adaptive response to challenge and generally unharmful [67], long-term secretion of the cortisol is considered to play a crucial role in mediating the link between chronic stress and negative effects on physical and mental health [68]. Given the possible negative health effects of chronic long-term stress, in this study we use a new non-invasive, easily conducted and well tolerated method of analysis of cortisol from hair samples. Three-cm segment of hair is collected by a trained person with small scissors as close to scalp as possible, according to a validated protocol [69]. Hair samples are taken from the posterior vertex region in accordance with guidelines published by the *Society of Hair Testing* [70]. Considering that hair grows approximately 1 cm per month [71], hair samples are obtained in order to evaluate hair cortisol levels representative of the last 3 months [72]. Each sample is wrapped in aluminum foil to protect it from light and humidity and stored in closed plastic bags (marked with the ID, date and initials of laborant) at room temperature. Later, the samples will be analyzed at the Dresden University of Technology, Germany using an Enzyme-Linked Immunosorbent Assay (ELISA), a reliable and valid method to assess hair cortisol levels, which is highly positively correlated with liquid chromatograph–mass spectrometry (LC–MS/MS) [73].

### Cognitive assessment

The neurocognitive session includes five tests assessing memory, language and executive function:

#### Rey auditory verbal learning test (RAVLT) [74]

RAVLT is a powerful neuropsychological tool that is used for assessing episodic memory by providing scores of evaluating different aspects of memory including immediate recall, short-term and long-term memory, and verbal learning. The participant is required to learn a list of 15 words presented verbally at 2-s intervals over the course of 5 trials and recall verbally as many of the words as possible in any order after each trial. After approximately 30 min participants are asked to recall the words without repeated presentation to assess the long-term memory. The RAVLT is sensitive to verbal memory deficits caused by variety of neurological conditions [75].

#### Verbal fluency test [76]

We use 2 measures of verbal fluency, phonemic and semantic. Participants are asked to recall verbally as many words beginning with S, K and P (phonemic fluency) and as many animal names (semantic fluency) as they can. Sixty seconds is allowed for each of the 4 tests. Verbal fluency is used to investigate the presence of cognitive impairment, neurodegenerative and psychiatric conditions [77].

Three computerized tests from *NIH-Examiner battery* developed to reliably and validly assess domains of executive function [78]: Flanker Attention test, Set Shifting task, and Nback and 2Nback tests. Tasks are applicable to a broad range of individuals from different age and ethnic groups. Flanker task is a response inhibition task used to assess the ability to suppress responses that are inappropriate in a specific context; Set shifting task assesses cognitive flexibility and Nback and 2Nback assess working memory. During these tasks frontal brain structures are thought to be activated [78] . We used back translation method to translate the tests into the Czech language. Each participant completes these 3 tasks on laptops in the Psychopy program (version 1.73) [79].

### Neuroimaging

Scanning is carried out at the Department of Human Movement Studies, University of Ostrava, using a 1.5 T Siemens Magnetom Sempra Scanner with a 16- channel head coil (1.5 T Siemens Magnetom Sempra Scanner; Siemens, Erlanger, Germany). The neuroimaging protocol comprises structural sequences only, lasting approximately 10 min. MRI sequences include high-resolution T1-weighted, T2-weighted and 3-scan trace diffusion-weighted imaging. A full description of the MRI parameters adopted in our sequences is provided in Table 2. Cortical surface reconstruction and subcortical segmentation is completed via FreeSurfer (version 6.2), including total grey and white matter as well as subcortical volumes, cortical thickness and cortical surface area estimates for cortical regions [80]. Images undergo quality control prior to processing by 3 experts: a radiologist with 10 years of experience and two image analysts, each with 2 years of experience. Brain tissues and subcortical regions are visually inspected to ensure an accurate segmentation, gauge the severity of motion, intensity homogeneity, white matter underestimation, pial overestimation and artifacts [80]. Quality control of FreeSurfer reconstructions is based on MELD Protocol 3 [81].

**Table 2:** Set up of the MRI protocol used in the study

MRI sequences and parameters				
Imaging parameter	3D T1 MPRAGE	Axial T2 TSE	Axial T2 Dark fluid TSE FS	ep2d diff 3scan trace
Repetition time (ms)	2200	3200	7910	4800
Echo time (ms)	2.72	91	83	113
Inversion time (ms)	900	–	2359	–
Slice thickness (mm)	1	4	4	5
Field of view (mm)	256	230	215	230
Matrix size	197 × 256	210 × 320	179 × 256	176 × 176
Flip angle (°)	8	150	150	–
Bandwidth (Hz/Px)	160	191	190	812
Echo train length	–	7	4	–
Signal average	1	2	2	–
Number of slices	192	34	34	20
Acquisition time (min:s)	3:39	1:38	2:24	1:23

*MPRAGE (Siemens)* Magnetization Prepared – Rapid Gradient Echo; *TSE* Turbo Spin Echo, *FS* Fat Saturation

### Long-term monitoring and EMA protocol

Following baseline assessments, participants are monitored for 12 months using the Fitbit Charge 3 monitor. Additionally, participants complete brief surveys administered on their smartphones through a mobile app developed for the study. The intensive assessment incorporates elements of ecological momentary assessment (EMA) and will be administered four times for 2 weeks across the 12-month period (at baseline, month 4, month 8, and month 12). In an EMA study, participants are usually prompted several times during the day or are asked to self-initiate a report when an event occurs. This largely reduces the risk of retrospective recall bias associated with summative judgements or recollection of behavior patterns [61]. EMA performed on smartphones has a number of advantages including automatic recording of the time stamp of answers in near-real-time, possibility of tracking of compliance and response patterns, and possibility of combining survey responses with other data such as those from a connected monitoring device (here the Fitbit Charge 3 monitor) or other data from the smartphone device or from other online sources.

In this study, participants complete four 14-day bursts of EMA data collection. Four pseudo-random surveys (sampling four daily periods: 8–11:59, 12–15:59, 16–19:59, 20:00–22:00) assess momentary variability in affective states, stress, pain, and context. The morning survey includes questions about sleep and the evening survey (last of the day) also includes some retrospective end-of-day assessments. Additionally, participants are asked to complete two types of self-initiated reports: (1) PA report (episodes of intentional PA lasting at least 20 min and leading to increased heart rate, breathing and sweating) and (2) injury report (when encountering injury associated with physical activity). Every Sunday between 16:00 and 20:00 participants also receive a weekly injury survey (to facilitate prospective monitoring of injury incidence even in cases when a self-initiated injury report may not have been filed). This weekly survey was also enriched by COVID-19 related questions during the pandemic restrictions to evaluate factors associated with PA and mental health (for details please see description at https://www.covidminds.org/longitudinal-studies; The 4HAIE study). Finally, a context-trigged survey is initiated when Fitbit data indicate a decrease in PA in a given week below 90% confidence interval of the average level of the past 4 weeks. This floating algorithm allows us to detect potential periods of decline in PA that may also be indicative of incurred injury.

**Fig. 2:**
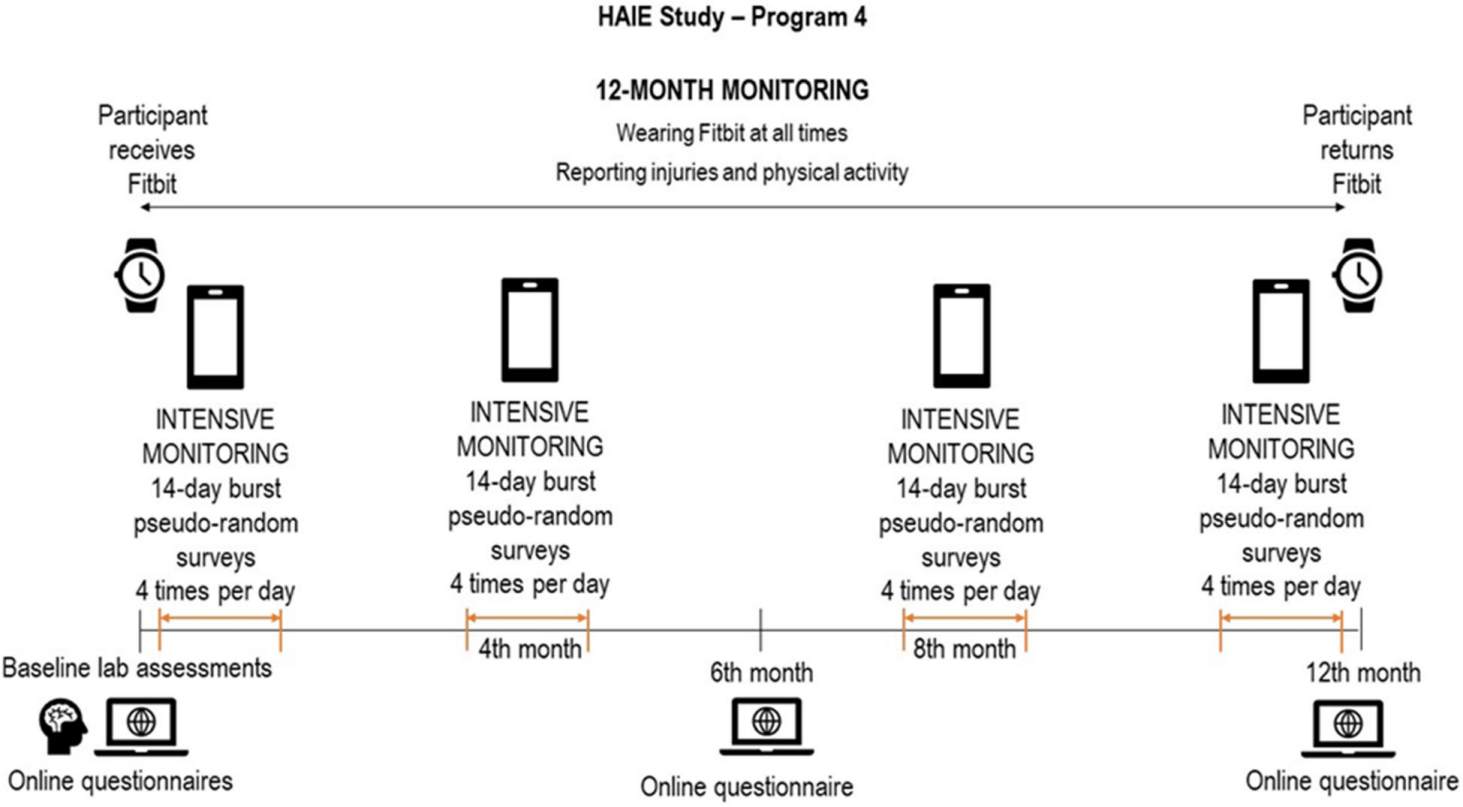
Graphic overview of the study protocol. *Note*. Figure adapted with permission from the Czech Information System on Air Quality (ČHMÚ). Data are from 2018 and reflecting limit value valid from 2020

In this study, we also collect real-time data about air quality from a network of available public air quality measurement stations. A server application developed for the study specifically aggregates publicly available data on all available air pollution parameters (data downloaded every 30 min) and integrates them with GPS data obtained from the smartphone while protecting participant privacy. Exact GPS location of participants is not recorded, only data from the linked nearest public air quality monitoring station is used each time they make a report of an episode of PA or at least once per day when no activity is reported. Monitoring and combining these different streams of data will allow us to evaluate dynamically the associations among air pollution parameters, behavior and wellbeing, and so gain rich insight into patterns of healthy functioning and within-person fluctuations of variables of interest across moments, days, or weeks. Figure 2 depicts the overview of the entire study protocol including the EMA bursts.

## Discussion

Within this protocol, we introduce a unique interdisciplinary study to longitudinally assess the associations among air pollution, physical (in) activity, running-related injuries and psychosocial factors in a sample of *n* = 1500 participants across the adult lifespan (18–65 years). As part of the study, rich laboratory assessments are conducted (on physiological, biomechanical, anthropometric, cognitive and neuroimaging variables) in combination with online surveys (sociodemographic, psychological, physical activity, biomechanical variables) to establish baseline characteristics of the study cohort. These cross-sectional assessments will be followed with long-term follow-up assessments 5 years later (as part of a follow-up project) to allow for detection of risk/protective factors in the context of long-term change. More acute, short-term effects of air pollution and PA status on sleep, psychological and injury parameters can also be evaluated due to the dynamic 12-month monitoring (following baseline assessments) using objective sensory data (Fitbit) and mobile apps. These intensive longitudinal data will enable prediction of *individual trends* in sleep, physical activity, injury risk, and air pollution to help establish suitable future intervention targets.

It is of particular interest in this study to evaluate and detect important predictors of running-related injuries (RRI), including potential differences in incidence rates by air-pollution or PA status. To this end we collect prospective weekly self-report data as well as self-initiated in-time reports of PA-related injuries via the study mobile app. To this date (October 2020), 287 self-initiated reports of occurred injuries have been received from *n* = 171 participants. In weekly reports, 396 individual reports of injuries have been collected from *n* = 261 participants. We have received 7777 at least partially answered PA decrease surveys from *n* = 844 participants (additional 1691 surveys were returned unanswered). These data can be aggregated with other EMA and air pollution data to assess whether there are differential rates of injuries across air pollution and PA status and what psychological, biomechanical, or physiological factors may underlie the differences. There is a lack of RRI studies with dynamic EMA psychosocial data in the literature. Some studies suggest that psychosocial variables can be predictors for RRI. For example, scores on self-reported mental health and negative affect were predictive of injury status in another two-year prospective study, with runners who suffered injury reporting worse mental health status and more negative emotions [82]. In addition, dynamic EMA data from HAIE study may indicate whether variations in psychosocial variables may be serve as meaningful predictors of RRI. This information may aid in future interventions aimed at maintaining recommended levels of PA while minimizing running-related injuries.

Additionally, longitudinal Fitbit data combined with EMA survey data on the context of PA will allow us to evaluate PA decreases throughout the 12-month monitoring period, including decreases unrelated to injuries. While previous studies have focused on predicting the participation and levels of PA [83, 84], little attention has been devoted to studying for instance weekly or monthly fluctuations and decreases in PA. Although some level of PA is better than none and even dose lower than recommended may bring some health-related benefits [85], it is regular and sustained PA that is likely to garner the most positive effects on health and related variables. It is thus important to evaluate the predictors of decreases in PA and fluctuations specifically, to aid future interventions focused on the long-term maintenance of PA.

The integration of mental and neurocognitive assessments and brain MRI in our study, in combination with data on physiological and behavioural variables, may provide relevant knowledge about the association between air pollution and cognitive and brain health outcomes observed in many but not all studies [40, 42–46, 48, 51, 80]. While PA has been long considered a protective factor for dementia, air pollution has been acknowledged as one of the key risk factors for dementia only recently [86]. The interactions between level of PA and air quality have been therefore rather underinvestigated, supporting the importance of our study. Our findings may contribute to the small amount of literature examining the psychological and neurological benefits of regular exercise in non-polluted and polluted environments [57]. Furthermore, due to wide range of PA levels (particularly in the active group), the 4HAIE study may help to unveil whether the potential association between level of PA and mental and cognitive functioning is linear or not. There might be an optimal level of PA beneficial for the brain while extreme levels of PA might be rather ineffective or even detrimental to brain health.

The interdisciplinary approach of our study and multiple laboratory data collection, including blood markers, cardiac autonomic functioning, and brain MRI imaging, will enable us to investigate potential biological mechanisms linking air-pollution and PA status, to mental and cognitive health. We expect cognitive function and specific brain structures such as prefrontal cortex, white matter and hippocampus, to be lower and smaller in volume, respectively, in adults from air-polluted environment and in inactive controls. Our primary plausible physiological mechanisms underlying the association between air-pollution and worse mental and cognitive health are higher level of chronic inflammation and oxidative stress, lower level of BDNF, and cardiac and vascular functioning [52].

In a longitudinal perspective, based on laboratory data collected in future waves, this study may help to decribe psychological and behavioral factors affecting resilience against age-related changes in the brain, cognition or mental health. This hopefully will generate ways of protecting healthy cognitive and mental aging in air-polluted environment, an approach that is needed, given the current limited progress specifically in preventing dementia [86] and the large number of people chronically exposed to poor air quality [6].

### Anticipated challenges and study limitations

The timing of the study coincided with the COVID-19 pandemic which has presented a unique set of challenges with respect to the main study aims, but also some interesting opportunities. Whereas there is evidence that there have been intermittent reductions in air pollution in response to restrictions on mobility [87], air pollution has been linked with worse COVID-19 disease outcomes [7, 8]. The pandemic restrictive measures have also led many countries to take drastic restrictions to prevent the transmission of the virus such as limiting human interaction, quarantines, imposing a curfew and even locking down entire cities [88]. While this means that our study will uexpectedly allow us to naturally capture changes in behavior and psychological factors before, during, and after restrictive measures employed in the Czech Republic to combat the pandemic, it poses dilemmas in interpreting the study results. A number of reports have described how the pandemic-related restrictions impacted negatively population mental health [89] and at the same time, the imposed lockdowns have led to improved air quality, albeit intermittent [87, 90]. In our study, more specifically, in *n* = 557 participants at least one EMA burst but no more than three out of the four bursts occurred prior to the first set of restrictions, which came into force on March 12th, 2020. Out of the n = 557 participants, 175 participants had completed one EMA burst prior to the restrictions (29 out of these were undergoing their 2nd burst on the day of the first restrictions), *n* = 225 participants had completed two bursts (24 of these were undergoing their 3rd burst at that time), and *n* = 157 had completed three bursts. Thanks to the 12-month duration and ongoing enrollment of participants, the study is likely to continue past the end of restrictions of the second wave of the pandemic (Fall-Winter 2020), allowing us to capture the impact of variously strict restrictions as well as the return to the normal or “new normal” state of functioning.

The role of PA as a health-enhancing activity has been highlighted also as part of the ongoing COVID-19 pandemic, although ironically opportunities for PA have been reduced as a consequence of pandemic restrictions. Studies indicate that people who reported decreases in PA during COVID-19 mitigation strategies reported increased stress and anxiety levels [91–94]. For some groups of the population, the social distancing and isolation regulations during the pandemic may have put new barriers in engaging in PA [94]. Given that PA represents a potential coping strategy for mental health issues, our study may help shed light on how PA behavior has changed during the COVID-19 pandemic and what factors help predict sustained PA patterns.

The large sample size is a strength of this study. The recruitment strategy involves quota sampling to ensure predetermined distribution by age, gender, air pollution and PA status, still it relies on volunteers who own smartphones and have online access. These requirements precluded drawing a nationally representative sample. For example, based on descriptive data on the first two thirds of participants involved the 4HAIE study, there is about 45% of men and women with university degree while the average proportion of people with completed university degree is around 24% in the Czech Republic [95]. Given the fact that education is one of the greatest protective factors in mental and brain health, the potential association between air-pollution and mental and cognitive health outcomes and neural correlates may be underestimated due to the higher proportion of people from higher socioeconomic status in our study. Nevertheless, efforts are being made to recruit across social status and target individuals with lower education and income status, and, importantly, to ensure the distribution of the educational level in the 4HAIE cohort is similar across the regions and PA status to minimize impact of selection bias.

Given the interdisciplinary nature of the study, the most intriguing study hypotheses will lie in evaluating interactions among variables at different levels of analysis and across disciplines. Although having laboratory tests means we do not have to rely on self-reported information only, it led to rather extensive assessment batteries that pose high burden on participants. While dropout rates remain low and adherence statistics appear acceptable, future follow-up assessments are likely to be trimmed and streamlined. The large-scale nature of the study is ambitious especially with respect to the EMA data collection which relies on participants’ own devices. Although extensive preliminary work preceded deployment of our mobile app and server, ongoing technical issues persist for certain types of devices. This may contribute to the challenge of maintaining optimal response rates in the EMA part of the study and missing data management. The participants receive payment for their baseline laboratory assessments and are eligible to win in a monthly lottery, nonetheless, there are no other explicit incentives to continue within the study and complete the EMA and weekly surveys. With 661 participants past the 6-month study mark, the average completion rate for the momentary EMA surveys is around 70% with response rates for weekly surveys around 76%. When aiming to maximize compliance in EMA studies, a fine balance must be struck between providing sufficiently motivating extrinsic incentives (e.g., renumeration) and biasing data with too high of an incentive [96]. Intrinsic incentives offer an alternative approach and can be incorporated into existing technologies with advanced features such as elements of gaming or provision of feedback on response rates within the study [97–99]. In the 4HAIE study, we employ ongoing surveillance of study adherence with a system of both automatic notifications and reminders (app) as well as telephone, SMS, and email check-ins and follow-up contacts when necessary. Our approach was designed to align with recommended strategies for optimizing response rates in EMA studies such as incorporating ongoing compliance monitoring, check-in strategies and providing compliance-based incentives to support participant engagement [99, 100].

## Conclusion

The 4HAIE study is an interdisciplinary investigation of both long-term and short-term (acute) associations among air pollution, physical (in) activity, sleep, running-related injuries and psychosocial factors across the adult lifespan. Rich laboratory assessments are combined with online surveys and dynamic 12-month monitoring. The study cohort will be followed prospectively (in 5 years) beyond this 12-month period, allowing for the assessment of individual risk factors as well as long-term patterns in physical activity, sleep, injury risk, and air pollution to help establish suitable future intervention targets. Future results of this large-scale study may also serve to underscore the importance of air pollution prevention and mitigation measures to policymakers in the Czech Republic (especially in the highly polluted region).

## Data Availability

Not applicable.

## Abbreviations

HAIE: Health Aging in Industrial Environment
4HAIE: Health Aging in Industrial Environment - Program 4
WHO: World Health Organisation
PM_10_, PM_2.5_: Particulate matter
NO_2_: Nitrogen dioxide
CO: Carbon monoxide
SO_2_: Sulphur dioxide
AQI: Air quality index
PA: Physical activity
MSR: Moravian-Silesian Region
SBR: South Bohemian Region
MRI: Magnetic resonance imaging
CNS: Central nervous systém
HR: Hazard ration
BDNF: Brain-derived neurotrophic factor
DXA: Dual-energy xray absorptiometry
DREAM: Dynamic Real-time Ecological Ambulatory Methodologies
EMA: Ecological momentary assessment
RRI: Running-related injuries
